# 230 Optimising Outdoor Recreational Activities in Bali, Indonesia: The Significance of the Cultural Landscape Subak as a Health-Enhancing Environment

**DOI:** 10.1093/eurpub/ckae114.092

**Published:** 2024-09-26

**Authors:** Kei Nilsson

**Affiliations:** World Health Organization Regional Office for Europe, Copenhagen, Denmark; Swedish University of Agricultural Sciences, Alnarp, Sweden

## Abstract

**Purpose:**

Subak – the terraced rice landscapes of Bali – appears to be more protected and better preserved than the rest of the greenspaces in Indonesia. As one of the first studies of outdoor recreational activities in the cultural landscape subak, this study aims to describe the significance of this environment – which is also a UNESCO World Heritage Site – for the activities to promote and improve health and well-being.

**Methods:**

The study compiled a quantitative questionnaire answered by fifty-eight respondents, five semi-structured in-depth interviews, and a series of participant observations.

**Results:**

Based on mixed methods analyses, the results revealed: (1) outdoor recreational activities in the subak areas have yielded benefits, namely improved cognitive and social skills, as well as an enhanced state of health and well-being, (2) the role of the subak areas in facilitating outdoor recreational activities has shown cognitive, emotional, and behavioural changes in the study participants, and (3) outdoor recreational activities in the subak areas have fostered a more environmentally conscious mindset that helps improve human health and well-being.

**Conclusions:**

This study elucidated possible implications that underlined the activities in the subak as a way to adopt pro-environmental behaviour (PEB) and thereby helped improve human health and well-being, protect the subak environment, and achieve the Sustainable Development Goals (SDGs) in the context of this study, particularly SDG 3 Good Health and Well-Being, SDG 11 Sustainable Cities and Communities, and SDG 12 Responsible Consumption and Production. Overall, the mixed methods analysis applied in the study provided new transcendent perspectives that in future research can form the starting point to emphasise the significance of the greenspaces, namely the cultural landscape subak, as a health-enhancing environment for outdoor recreational activities to promote and improve health and well-being.

**Funding:**

The fieldwork was funded by the Swedish government Sida (Swedish International Development Cooperation Agency) through a scholarship of 27,000 SEK.

